# Radiofrequency Ablation for Hemorrhoidal Disease

**DOI:** 10.3390/life16061025

**Published:** 2026-06-18

**Authors:** Eremeev Spiridon, Cristian Ichim, Paula Anderco, Ciprian Tanasescu

**Affiliations:** Faculty of Medicine, Lucian Blaga University of Sibiu, 550169 Sibiu, Romania; spiridon.eremeev@ulbsibiu.ro (E.S.); ciprian.tanasescu@ulbsibiu.ro (C.T.)

**Keywords:** radiofrequency ablation, internal hemorrhoids, rafaelo procedure, hemorrhoidal disease

## Abstract

Hemorrhoidal disease is a common anorectal condition that may require treatment when bleeding, prolapse or persistent symptoms fail to respond to conservative or office-based therapy. Radiofrequency ablation (RFA) has emerged as a minimally invasive, tissue-sparing technique for symptomatic internal hemorrhoids, based on controlled delivery of high-frequency energy into hemorrhoidal tissue. The resulting thermal effect induces coagulative necrosis, fibrosis, mucosal fixation and progressive reduction in hemorrhoidal volume, without excisional removal of anoderm or rectal mucosa. This narrative review summarizes the mechanism, technical principles, clinical advantages, comparative evidence and remaining uncertainties surrounding RFA, with particular attention to the Rafaelo procedure and related radiofrequency-based approaches. Current evidence suggests that RFA may reduce postoperative pain, analgesic requirements, wound-related morbidity, hospital stay and time to return to normal activity compared with conventional hemorrhoidectomy, while maintaining acceptable short- and mid-term symptom control in selected patients, especially those with grade II–III internal hemorrhoids. However, available studies remain heterogeneous in design, technique, patient selection, outcome definitions and follow-up duration. The relationship between modern probe-based RFA and earlier radiofrequency-based approaches, including Ellman surface coagulation, Celon bipolar radiofrequency-induced thermotherapy and radiofrequency-assisted hemorrhoidectomy, remains insufficiently standardized in the literature. Further randomized trials, standardized outcome reporting, long-term recurrence data and cost-effectiveness analyses are required to define the optimal indications and therapeutic position of RFA.

## 1. Introduction

Hemorrhoidal disease is among the most common anorectal conditions encountered in surgical practice and represents a frequent cause of consultation in both outpatient and operative settings [1,2]. Although most patients can be managed with conservative treatment or office-based procedures, a clinically relevant subset ultimately requires procedural or surgical intervention because of persistent bleeding, symptomatic prolapse, advanced disease or failure of previous therapies [3].

Treatment selection is primarily guided by symptom severity, the degree of prolapse, patient preference and the risk–benefit profile of each intervention [4,5]. According to the Goligher classification, surgical treatment is generally considered for grade III–IV hemorrhoids, whereas selected grade II cases may require escalation when symptoms persist despite conservative or office-based treatment [6]. Conservative measures, including dietary optimization, fiber supplementation, lifestyle modification and bowel-habit regulation, are usually recommended for milder disease, while office-based procedures such as rubber band ligation, infrared coagulation and sclerotherapy are commonly used for persistent symptomatic internal hemorrhoids [7,8,9]. More advanced or refractory disease may require interventions such as hemorrhoidal artery ligation, conventional hemorrhoidectomy or stapled hemorrhoidopexy [10,11,12].

Conventional excisional hemorrhoidectomy, performed either through the open Milligan–Morgan technique or the closed Ferguson approach, remains one of the most effective treatments for controlling bleeding and prolapse, with durable symptomatic relief and low recurrence rates [13]. However, this effectiveness is counterbalanced by postoperative pain, delayed functional recovery and procedure-related morbidity, including urinary retention, anal stenosis, bleeding and continence disturbances [14]. These limitations have driven the search for less invasive techniques that preserve anorectal anatomy while reducing postoperative discomfort and recovery time.

Radiofrequency ablation (RFA) has therefore emerged as a promising minimally invasive option for hemorrhoidal disease, including symptomatic internal hemorrhoids, with the potential to provide shorter treatment, reduced postoperative pain and faster recovery compared with conventional surgical approaches [15]. The technique is based on the delivery of radiofrequency electrical energy, commonly at 4 MHz, through a dedicated probe inserted into the hemorrhoidal tissue [16]. This targeted energy delivery produces controlled thermal injury, leading to coagulative necrosis of hemorrhoidal vascular tissue, followed by fibrosis, tissue contraction, mucosal plication, reduction in hemorrhoidal volume and symptomatic improvement [17,18].

Among radiofrequency-based techniques, the Rafaelo procedure is one of the most widely reported approaches [19]. It is designed as a tissue-sparing intralesional treatment and can be performed under local anesthesia, with or without sedation, thereby potentially reducing anesthesia-related risk and facilitating outpatient management [17,20]. Several studies have reported favorable outcomes after RFA, including low postoperative pain, rapid recovery, high patient satisfaction and acceptable complication rates [16,21,22,23]. Nevertheless, the current evidence base remains less mature than that supporting established treatments and direct comparative data between RFA, office-based procedures and conventional hemorrhoidectomy remain limited. Therefore, this review aims to evaluate the therapeutic role of RFA in internal hemorrhoidal disease, with particular emphasis on its mechanism, clinical advantages, comparative outcomes and remaining evidence gaps.

## 2. Hemorrhoidal Disease and Current Therapeutic Landscape

Hemorrhoids are normal anatomical structures composed of vascular tissue, connective tissue, smooth muscle fibers and overlying mucosa, which contribute to fine continence and protection of the anal canal during defecation [24,25,26]. Hemorrhoidal disease develops when these cushions become symptomatic as a result of vascular engorgement, structural degeneration of supporting connective tissue, inflammatory changes and progressive distal displacement of the anal cushions [27].

Internal hemorrhoids arise above the dentate line and are therefore primarily covered by columnar or transitional mucosa, whereas external hemorrhoids originate below the dentate line and are covered by anoderm [28,29]. This anatomical distinction is clinically important because internal hemorrhoids are less pain-sensitive unless complicated by thrombosis, strangulation or associated anorectal pathology, while external disease is more frequently associated with pain and local discomfort [30].

The most widely used classification for internal hemorrhoids is the Goligher classification, which stratifies disease severity according to the presence and reducibility of prolapse (Table 1) [31,32]. Grade I hemorrhoids bleed without prolapse, grade II hemorrhoids prolapse during defecation but reduce spontaneously, grade III hemorrhoids require manual reduction and grade IV hemorrhoids remain irreducibly prolapsed [6]. Although this classification remains useful for therapeutic orientation, it does not fully capture symptom burden, hemorrhoidal volume, number of affected cushions, mixed internal–external disease or the impact on quality of life [33,34].

Clinical presentation is heterogeneous and may include painless rectal bleeding, prolapse, pruritus, mucus discharge, swelling, discomfort, soiling and pain, particularly when complications or external components are present [28,35,36]. Among these manifestations, bleeding and prolapse are usually the key symptoms guiding escalation from conservative management to office-based or surgical treatment [37]. Recurrent symptoms may lead to repeated consultations, impaired daily functioning, reduced quality of life and increased healthcare utilization, particularly in patients with persistent bleeding or advanced prolapse [32].

Management of hemorrhoidal disease should follow a stepwise, grade-oriented approach, beginning with conservative treatment and escalating according to symptom persistence, prolapse severity, external components, patient preference and procedural fitness (Table 2). Conservative treatment is recommended as first-line therapy across Goligher grades, whereas office-based procedures are mainly used for grade I–II hemorrhoids and selected grade III disease after failure of conservative measures. Surgical treatment is generally reserved for advanced, refractory or combined internal–external hemorrhoidal disease, particularly grade III–IV disease, while RFA may be considered only in selected symptomatic grade II–III internal hemorrhoids where tissue-sparing treatment and rapid recovery are priorities [38,39].

## 3. Radiofrequency Ablation: Mechanism and Technique

Radiofrequency ablation (RFA) is based on the controlled delivery of high-frequency electromagnetic energy into hemorrhoidal tissue, producing localized thermal injury rather than surgical excision [40,41,42]. In the literature, several related but not identical techniques are described, including radiofrequency thermocoagulation, RFA with the Rafaelo device and radiofrequency-assisted hemorrhoidectomy; therefore, the procedural terminology should be interpreted carefully when comparing outcomes across studies [43].

### 3.1. Biological Effects of Radiofrequency Energy

At the biological level, radiofrequency energy acts through a high-frequency alternating current that heats a volume of tissue around the active electrode and induces focal coagulative necrosis with limited injury to surrounding tissue. The thermal effect results from resistive heating within the treated tissue, because ionic agitation generated by the alternating current is converted into frictional heat. Temperature and exposure time determine the extent of tissue injury, with sustained heating above approximately 50 °C producing irreversible cellular damage and temperatures above approximately 60 °C producing rapid coagulative necrosis. If energy delivery is excessive, tissue desiccation and charring may occur, increasing local impedance and reducing further energy deposition into the target tissue [44].

In hemorrhoidal disease, this thermal effect is applied within the hemorrhoidal cushion to produce coagulation, tissue shrinkage and progressive reduction in hemorrhoidal volume rather than excisional removal of anoderm or rectal mucosa [45]. After the acute coagulative injury, the treated hemorrhoidal tissue undergoes edema, fibrosis and healing, which contribute to mucosal fixation and reduction in prolapse during follow-up [46]. These biological effects explain the clinical rationale of RFA: reduction in bleeding, reduction in prolapse, preservation of anodermal and mucosal bridges, and avoidance of an open excisional wound. Because radiofrequency energy produces heat, technical safeguards are required to reduce collateral injury, including submucosal local anesthetic infiltration, intralesional probe placement above the dentate line, visual monitoring of tissue blanching and local cooling after energy delivery [45,46].

### 3.2. Rafaelo Radiofrequency Ablation Technique

The Rafaelo procedure applies the same principle of radiofrequency-induced thermal injury, but uses a dedicated intralesional probe rather than a surface ball electrode [17]. After insertion of a lubricated proctoscope, the hemorrhoidal cushion is visualized and local anesthetic is injected into the submucosal plane around the target tissue [19]. This injection provides analgesia and creates a protective fluid barrier between the hemorrhoidal tissue and the internal anal sphincter, thereby limiting heat transmission to the sphincteric complex [16].

The HPR45i probe is then introduced directly into the hemorrhoidal cushion, usually above the dentate line and radiofrequency energy is applied through the probe tip [47]. Because the energy is delivered intralesionally, the thermal effect is concentrated within the hemorrhoidal tissue, allowing targeted coagulation while avoiding broad anodermal or rectal mucosal excision. During energy delivery, the tissue is monitored for blanching or whitish discoloration, which indicates sufficient thermal effect [17]. After ablation, the probe is withdrawn and the puncture site can be coagulated if needed to secure hemostasis [16]. Some protocols also apply cold saline-soaked gauze after treatment to cool the tissue surface and reduce residual thermal spread. The same sequence is repeated for each clinically relevant hemorrhoidal cushion, with final inspection to confirm hemostasis and exclude immediate bleeding.

### 3.3. Practical Procedural Considerations

RFA is commonly performed as a short procedure and may be carried out under local anesthesia, with or without sedation, depending on the protocol, patient tolerance, disease grade and institutional practice [46]. The procedure is particularly attractive because it avoids open wounds, preserves anodermal and mucosal bridges and aims to maintain the anatomical role of the anal cushions while reducing their pathological enlargement [25,26]. Postprocedural care usually includes simple analgesia, stool regulation, avoidance of straining and early follow-up to assess pain, bleeding, urinary symptoms and recurrence [48].

Overall, the technical rationale of RFA is tissue remodeling rather than tissue excision: controlled energy delivery produces necrosis, fibrosis and contraction of the hemorrhoidal cushion, with the aim of reducing symptoms while minimizing postoperative pain and preserving anorectal anatomy [46].

The optimal candidates for RFA appear to be patients with symptomatic internal hemorrhoids, particularly Goligher grade II–III disease, in whom bleeding or reducible prolapse persists despite conservative or office-based treatment and in whom tissue preservation, reduced postoperative pain and rapid recovery are important therapeutic priorities [16,17,46]. RFA is best considered when the dominant pathology is internal hemorrhoidal enlargement without major external components, fixed circumferential prolapse, strangulation or advanced mixed internal–external disease. Conversely, patients with grade IV hemorrhoids, large external disease, fixed prolapse, suspected malignancy, acute anorectal sepsis or conditions requiring definitive excisional correction are less suitable candidates for stand-alone RFA and should be assessed for conventional surgical management [39].

## 4. Advantages of Radiofrequency Ablation

RFA offers several potential advantages in hemorrhoidal disease because it is designed to achieve symptom control through controlled thermal remodeling rather than excisional tissue removal [15]. By delivering energy directly into the hemorrhoidal cushion, RFA induces coagulative necrosis, fibrosis and progressive tissue contraction, while preserving the surrounding anoderm and rectal mucosa as much as possible [23,43]. This tissue-sparing mechanism is particularly relevant in internal hemorrhoidal disease, where preservation of the anal cushions and minimization of anorectal trauma are central objectives of modern treatment [49,50].

One of the most consistently reported benefits of RFA is reduced postoperative pain. In contrast to conventional hemorrhoidectomy, which creates open wounds in a highly sensitive anatomical region, RFA limits the extent of tissue injury and applies energy above the dentate line, where sensory innervation is less intense [51,52]. Prospective clinical evidence on the Rafaelo procedure has shown low postoperative pain scores, limited analgesic requirements and sustained symptom improvement during follow-up, supporting its role as a recovery-oriented intervention for selected patients with symptomatic internal hemorrhoids [46]. This advantage is clinically important because postoperative pain remains one of the main factors limiting patient acceptance of conventional hemorrhoidal surgery.

Another relevant benefit is the feasibility of performing RFA in an outpatient or day-case setting [53]. The procedure may be carried out under local anesthesia, with or without sedation, which can reduce the need for spinal or general anesthesia and facilitate faster discharge [46]. This procedural profile may be especially advantageous for patients in whom rapid recovery, reduced anesthesia exposure, or avoidance of hospital admission is desirable [18]. Early clinical experience with the Rafaelo technique has also reported significant improvement in hemorrhoidal severity scores, minimal discomfort and early return to normal activity, reinforcing its potential value as a minimally invasive alternative in advanced hemorrhoidal disease [54].

From a safety perspective, RFA appears to have an acceptable complication profile, although it should not be presented as risk-free. Reported adverse events are usually minor or manageable, including transient bleeding, discomfort, urinary symptoms or local thrombosis, while major complications such as anal stenosis, infection or continence impairment appear uncommon in the available series [55]. However, the heterogeneity of study designs, follow-up duration and definitions of complications limits the strength of conclusions regarding long-term safety. Therefore, the safety advantage of RFA should be interpreted as promising but still dependent on careful patient selection, technical standardization and adequate follow-up [56].

Despite these advantages, RFA has several relevant disadvantages that should be acknowledged. First, the available evidence remains limited by small sample sizes, heterogeneous techniques, mostly non-randomized designs, variable follow-up duration and the absence of large randomized controlled trials, which restricts the strength of conclusions regarding durability, recurrence and comparative effectiveness [23,47,57]. Second, symptom persistence or recurrence may occur after RFA and some patients may require repeat treatment or conversion to another office-based or surgical procedure. Third, because the technique depends on controlled thermal injury, inappropriate energy delivery or inadequate tissue protection may theoretically increase the risk of pain, bleeding, thrombosis, mucosal ulceration, local necrosis or collateral thermal damage. Fourth, RFA may be less suitable as a stand-alone treatment for fixed grade IV prolapse, large external components or circumferential prolapse, where excisional or reconstructive procedures may provide more definitive anatomical correction. Finally, cost, device availability, operator experience and lack of standardized treatment parameters may limit generalizability across healthcare systems.

Comparative evidence also supports the recovery-related advantages of radiofrequency-based treatment [58,59]. In randomized data comparing in situ RFA with Milligan–Morgan hemorrhoidectomy, radiofrequency treatment was associated with shorter operative time, shorter hospitalization, reduced post-defecation pain, faster wound healing and earlier return to work [60]. Similarly, RFA combined with plication showed lower postoperative pain, shorter hospital stay, faster healing and quicker return to activity than Milligan–Morgan hemorrhoidectomy in grade III hemorrhoids [61]. These findings suggest that the main comparative strength of radiofrequency-based approaches lies in reducing early postoperative morbidity rather than necessarily replacing more definitive excisional procedures in all patients.

The potential benefit of radiofrequency energy is further supported by studies in which radiofrequency instruments were used during hemorrhoidectomy. Radiofrequency hemorrhoidectomy has been associated with less severe postoperative pain and improved wound healing compared with conventional diathermy Milligan–Morgan hemorrhoidectomy [40]. In Ferguson hemorrhoidectomy, the use of a radiofrequency scalpel shortened operative time and reduced early postoperative pain compared with classic diathermy, supporting the concept that radiofrequency energy may limit early nociceptive burden when compared with standard electrosurgical techniques [62]. Although these studies do not evaluate Rafaelo-type intralesional RFA directly, they strengthen the broader biological rationale that controlled radiofrequency energy may reduce collateral thermal injury and postoperative discomfort.

The available clinical evidence supporting radiofrequency-based treatment is heterogeneous and includes studies of intralesional RFA, radiofrequency thermocoagulation, radiofrequency-assisted hemorrhoidectomy and selected comparator procedures. These data are summarized in Table 3 to contextualize the clinical positioning of radiofrequency-based techniques rather than to provide a separate analysis of non-radiofrequency alternatives.

Nevertheless, RFA should be positioned as a promising but still evolving technique, rather than as an established replacement for all conventional treatments. Available follow-up after Rafaelo-type RFA is generally short to intermediate, most commonly ranging from 6 to 12 months, although one prospective two-center study reported outcomes up to 24 months [16,17,46]. In the systematic review of the Rafaelo procedure, all included studies were non-randomized, and recurrence and reoperation were reported but the overall certainty of evidence was rated as very low [23]. In the UK case series, patients were followed for 6–12 months and five patients required further operative management during this period, with recurrent symptoms being generally reported as milder than baseline [16]. In the French multicenter prospective study, 129 patients with mainly grade II–III hemorrhoids were followed for 1 year, seven patients (5.4%) required hemorrhoidectomy for relapse and the authors emphasized the need for longer-term randomized studies [17]. In another study of third-degree hemorrhoids, follow-up was available in 95% of patients at 6 months, 86% at 12 months and 74% at 24 months and 13 patients (12.7%) required repeat hemorrhoid therapy during the 2-year follow-up period [46]. These findings suggested that early recovery and symptom improvement after RFA are favorable, but long-term durability remains insufficiently defined because recurrence definitions, follow-up schedules and retreatment thresholds differ across studies. Compared with RFA, conventional hemorrhoidectomy appears more durable for advanced prolapse but is associated with greater postoperative morbidity. Selected office-based procedures are mentioned only as contextual comparators because they appear in the available comparative literature, not as separate alternatives analyzed in this review [70,71]. Further evidence is needed to clarify long-term recurrence, cost-effectiveness, optimal indications and comparative performance of radiofrequency-based techniques, particularly in relation to conventional excisional surgery and earlier RF approaches. Selected non-radiofrequency procedures are retained in Table 4.

## 5. Evolution of Radiofrequency-Based Techniques in Hemorrhoidal Disease

Despite its widespread use, the Goligher classification has important limitations as a therapeutic guide. It does not adequately incorporate mixed internal and external hemorrhoidal disease, nor does it reflect the number, volume or anatomical distribution of hemorrhoidal cushions [75,76]. Consequently, patients categorized within the same grade may differ considerably in clinical presentation, disease burden and expected response to treatment [38]. This heterogeneity complicates direct comparisons between interventions and may partly explain variations in reported efficacy and adverse event profiles. In addition, available guidelines are not entirely uniform and may differ in emphasis, scope, or therapeutic recommendations [77]. Therefore, clinical decision-making often integrates published evidence, guideline recommendations, local healthcare structures, cultural expectations regarding treatment and the clinician’s accumulated procedural experience [78].

Because radiofrequency-based treatment has been described using heterogeneous terminology and different devices, it is important to distinguish between earlier RF approaches and modern probe-based intralesional RFA. Earlier techniques included surface radiofrequency coagulation, non-excisional radiofrequency ablation combined with plication or fixation, bipolar radiofrequency-induced thermotherapy and radiofrequency-assisted hemorrhoidectomy. These approaches differ in energy delivery, depth of tissue effect, need for plication or excision and intended therapeutic goal. Current guidelines and guidance documents do not assign a uniform role to radiofrequency-based techniques in hemorrhoidal disease management (Table 5) [36,38,39,48,57,79].

### 5.1. Ellman-Based Surface Coagulation and Non-Excisional RF Techniques

Early Ellman-based radiofrequency techniques represented some of the first attempts to use high-frequency energy for hemorrhoidal disease. Ambulatory radiofrequency coagulation was initially described mainly for grade I–II bleeding internal hemorrhoids and used a ball electrode connected to a 4 MHz Ellman radiofrequency generator [80,81,82]. The electrode was applied to hemorrhoidal tissue to induce blanching, shrinkage and superficial coagulative necrosis while attempting to avoid excessive desiccation or charring [44,83]. This technique was attractive because it could be performed in an outpatient setting and was associated with limited postoperative discomfort, but its effect was primarily superficial and therefore more suitable for bleeding hemorrhoids than for advanced prolapse.

For more advanced prolapsing hemorrhoids, Ellman-based radiofrequency energy was combined with plication or fixation of the treated hemorrhoidal tissue [80,81,84]. In these non-excisional approaches, radiofrequency ablation was used to induce tissue shrinkage and coagulative necrosis, while plication or fixation aimed to correct prolapse and restore mucosal support [80,81,85]. Early clinical series reported symptom improvement, reduced postoperative pain, shorter hospitalization and faster return to activity compared with conventional hemorrhoidectomy [62,80,81,84,85]. However, these techniques were more operator-dependent than simple surface coagulation, required additional suturing or fixation and were not equivalent to modern intralesional probe-based RFA.

Technical difficulties included controlling the depth of thermal injury, avoiding mucosal ulceration or charring, ensuring adequate fixation of prolapsing tissue and standardizing energy delivery across hemorrhoidal cushions [47,57]. Comparative data suggested favorable early recovery compared with excisional procedures, but the evidence was mostly based on limited-center experience and did not establish a widely adopted standard technique [23,47,57].

### 5.2. Bipolar Intralesional RF Thermotherapy: The Celon Experience

Celon-based bipolar radiofrequency-induced thermotherapy differed from Ellman surface coagulation because energy was delivered through a bipolar applicator inserted into the hemorrhoidal tissue [20]. The technique was developed mainly for advanced grade III–IV hemorrhoidal disease and aimed to reduce blood flow within the hemorrhoidal cushion, induce tissue shrinkage and retract prolapsed hemorrhoids while preserving the anoderm and mucosa [20,41,57]. Its intratissue mode of application makes it conceptually closer to modern probe-based RFA than surface coagulation, although the devices, energy-delivery parameters and procedural workflow differ [17,46].

Early Celon/RFITT experience reported feasibility, symptom improvement and acceptable complication rates, particularly in patients with advanced prolapsing hemorrhoids [20,41,86,87]. However, the technique required accurate applicator placement, visual control, repeated applications and careful limitation of thermal spread. Technical challenges included maintaining the correct depth of insertion, avoiding injury below the dentate line, preventing excessive mucosal necrosis and achieving reproducible tissue contraction without overtreatment [88].

Comparative data remain limited and the technique has not been incorporated as a dominant guideline-based standard. Its current relevance is therefore mainly historical and conceptual: it illustrates the transition from surface radiofrequency coagulation toward intralesional tissue-remodeling strategies [84,87].

### 5.3. Radiofrequency-Assisted Hemorrhoidectomy

Radiofrequency-assisted hemorrhoidectomy, including the use of a radiofrequency scalpel or radiofrequency bistoury, should be distinguished from intralesional RFA [89]. In this setting, radiofrequency energy is used as an excisional or cutting–coagulating instrument during hemorrhoidectomy rather than as a stand-alone tissue-sparing ablation technique [85]. The therapeutic goal remains removal of hemorrhoidal tissue, but radiofrequency instruments may reduce lateral thermal spread, bleeding and early postoperative pain compared with conventional diathermy [88,89,90].

Comparative studies of radiofrequency hemorrhoidectomy have reported shorter operative time, reduced early postoperative pain and improved wound healing in some settings, including comparisons with conventional diathermy Milligan–Morgan or Ferguson hemorrhoidectomy [89,90]. Nevertheless, because radiofrequency-assisted hemorrhoidectomy remains an excisional procedure, it does not eliminate wound-related morbidity and should not be considered equivalent to Rafaelo-type intralesional RFA.

Earlier RF approaches show a gradual technical evolution from surface coagulation to non-excisional ablation with fixation, bipolar intralesional thermotherapy and RF-assisted excisional surgery [23,47,57,84,87]. Modern Rafaelo-type RFA differs from these methods by using a dedicated intralesional probe, standardized submucosal protection and a tissue-remodeling objective without hemorrhoidal excision. The evidence supporting earlier RF techniques however remains heterogeneous and many reports are limited by non-randomized design, variable terminology, inconsistent outcome reporting and incomplete long-term follow-up.

## 6. Limitations and Future Directions

Although RFA appears promising for internal hemorrhoidal disease, its current evidentiary base should be interpreted cautiously, because contemporary guidelines still rely mainly on established office-based and surgical procedures, while newer energy-based approaches require stronger comparative validation [39].

A major limitation in hemorrhoidal-disease research is the heterogeneity of reported outcomes, as previous interventional studies have used numerous and inconsistently defined endpoints, making direct comparison and meta-analysis difficult [91]. Future RFA trials should therefore adopt standardized outcome reporting, including the European Society of Coloproctology Core Outcome Set for hemorrhoidal disease, to improve comparability across studies [92].

The design of future RFA studies should follow the model of large pragmatic randomized trials in hemorrhoidal disease, as demonstrated by the HubBLe trial, which combined clinical outcomes with recurrence and health-economic evaluation [93]. Patient-reported outcomes should also be incorporated systematically, because validated instruments such as PROM-HISS capture symptoms, daily-life impact and satisfaction more directly than clinician-defined anatomical outcomes alone [94].

Long-term durability and cost-effectiveness should be prioritized, since the eTHoS trial showed that early postoperative advantages may not necessarily translate into superior long-term clinical or economic outcomes [95]. Future research should also clarify the ideal candidates for RFA according to Goligher grade, predominant symptom, hemorrhoidal volume, previous treatment history and patient preference, rather than treating internal hemorrhoidal disease as a uniform entity [39].

## 7. Conclusions

Radiofrequency ablation represents a promising minimally invasive option for the treatment of internal hemorrhoidal disease, particularly in patients with symptomatic grade II–III hemorrhoids who require escalation beyond conservative or office-based therapy. Its main advantages include tissue preservation, reduced postoperative pain, feasibility in an outpatient or day-case setting, rapid recovery and an acceptable safety profile.

Current evidence suggests that modern probe-based RFA should be interpreted within the broader evolution of radiofrequency-based hemorrhoidal treatment, progressing from surface coagulation and non-excisional ablation with plication or fixation to bipolar intralesional thermotherapy and radiofrequency-assisted hemorrhoidectomy. However, the available literature remains heterogeneous, with differences in technique, patient selection, outcome definitions and follow-up duration. Therefore, although RFA appears clinically valuable, it should not yet be regarded as a definitive replacement for established treatments.

Future well-designed randomized trials, standardized outcome reporting, longer follow-up and cost-effectiveness analyses are required to define the optimal indications, durability, and comparative role of RFA in modern hemorrhoidal disease management.

## Figures and Tables

**Table 1:** Goligher classification of internal hemorrhoids.

Grade	Defining Feature
I	Hemorrhoids bleed but do not prolapse
II	Hemorrhoids prolapse during defecation and reduce spontaneously
III	Hemorrhoids prolapse during defecation and require manual reduction
IV	Hemorrhoids are irreducibly prolapsed or immediately re-prolapse after reduction

**Table 2:** Management algorithm for hemorrhoidal disease according to Goligher grade.

Goligher Grade	Main Clinical Feature	Initial Management	Escalation if Symptoms Persist	Role of RFA
I	Bleeding without prolapse	Fiber, fluids, bowel-habit regulation, avoidance of straining, topical or pharmacological symptom control when appropriate.	Office-based treatment, mainly rubber band ligation, sclerotherapy, or infrared coagulation for persistent bleeding.	Not routinely first-line; may be considered only in selected refractory bleeding cases where energy-based treatment is locally available and clinically justified.
II	Prolapse during defecation with spontaneous reduction	Conservative treatment as first-line therapy.	Rubber band ligation is usually preferred; sclerotherapy or infrared coagulation may be considered depending on symptoms, availability, and patient preference.	Potential option in selected symptomatic grade II disease after conservative or office-based treatment failure, but evidence remains less mature than for established office procedures.
III	Prolapse requiring manual reduction	Conservative therapy followed by office-based treatment in selected patients, especially when prolapse is limited and not circumferential.	Repeat rubber band ligation, Doppler-guided hemorrhoidal artery ligation with or without mucopexy, stapled hemorrhoidopexy or excisional hemorrhoidectomy may be considered according to anatomy, symptom burden, and patient preference.	Most relevant potential indication for RFA, particularly in selected grade II–III internal hemorrhoids without dominant external disease, where tissue-sparing treatment and rapid recovery are priorities.
IV	Irreducible prolapse or immediate re-prolapse after reduction	Conservative measures may provide symptom relief but are rarely definitive.	Surgical treatment is generally recommended, with excisional hemorrhoidectomy favored for advanced prolapse or combined internal–external disease.	Not routinely recommended as a stand-alone definitive treatment; may be inappropriate when fixed prolapse or major external disease predominates.

**Table 3:** Summary of clinical evidence on radiofrequency-based and office-based treatments for internal hemorrhoids.

Intervention	Study Design	Population/Grade	Key Findings	Reference
Radiofrequency thermocoagulation	Retrospective	Hemorrhoidal disease, mainly grade III	RFT demonstrated clinical efficacy with good anatomical and symptomatic improvement; early minor complications were noted but manageable.	[55]
Radiofrequency ablation	Case series	Internal hemorrhoids, grade II–III	Minimally invasive technique with low postoperative pain and reduction in hemorrhoidal size; occasional persistence or recurrence observed.	[45]
Radiofrequency thermocoagulation	Retrospective	Symptomatic internal hemorrhoids	Significant reduction in bleeding and prolapse with improvement in quality of life; high patient satisfaction in long-term follow-up.	[22]
Pathophysiology/clinical features	Observational	Operated grade III–IV hemorrhoids	Highlights mechanisms: vascular congestion, inflammation, collagen alterations, and weakening of supporting tissue.	[63]
Rubber band ligation (RBL) vs. bipolar electrocoagulation	Randomized controlled trial	Grade II–III bleeding hemorrhoids	RBL showed higher success rates and required fewer sessions than bipolar coagulation, with similar recurrence at 1 year.	[64]
Radiofrequency thermocoagulation	Case series	Grade II–III hemorrhoids	Detailed procedural description; effective symptom control with low pain scores and rapid recovery.	[65]
Radiofrequency thermocoagulation	Retrospective	Grade II–IV hemorrhoids	Significant improvement in bleeding, prolapse, and QoL scores; high satisfaction and low analgesic requirements.	[21]
Endoscopic RBL	Randomized controlled trial	Grade I–III hemorrhoids	Comparable efficacy between ligation techniques; combined ligation associated with higher postprocedural pain.	[66]
Radiofrequency ablation	Prospective multicenter	Grade III hemorrhoids	Safe and effective with sustained symptom control over 2 years; low postoperative pain and outpatient feasibility.	[46]
Endoscopic RBL	Prospective observational	Grade I–III hemorrhoids	Effectiveness influenced by number of bands; higher band count improved outcomes in grade III disease.	[67]
Rubber band ligation	Retrospective	Grade II–III hemorrhoids	Safe, cost-effective outpatient procedure with significant symptom resolution and acceptable recurrence rates.	[68]
RFA + plication	Prospective	Advanced hemorrhoids (mainly grade III–IV)	Mechanism based on coagulative necrosis and tissue shrinkage; effective alternative to conventional hemorrhoidectomy.	[69]

**Table 4:** Selected comparative evidence used to contextualize radiofrequency-based treatment.

Comparison	Study Design	Hemorrhoid Grade	Main Procedural Differences	Main Outcome Differences	Ref.
Radiofrequency coagulation vs. rubber band ligation	Randomized prospective study	Grade II bleeding internal hemorrhoids	RFC uses 4 MHz radiofrequency energy to coagulate hemorrhoidal tissue; RBL mechanically strangulates the hemorrhoidal pedicle above the dentate line.	RFC was associated with less post-defecation pain, less tenesmus, and earlier return to activity, whereas RBL showed better hemorrhoidal obliteration and lower recurrence.	[72]
RFA vs. conventional hemorrhoidectomy	Retrospective propensity score-matched comparative study	Grade II–III internal hemorrhoids	RFA delivers 4 MHz energy through the Rafaelo probe, while hemorrhoidectomy involves excisional removal of hemorrhoidal tissue.	RFA had shorter operative time, less blood loss, lower opioid use, and better late postoperative pain control; hospital stay and complication rates were comparable.	[18]
Rubber band ligation vs. hemorrhoidectomy	Cost-effectiveness and cost-utility analysis from HOLLAND RCT	Grade III hemorrhoids	RBL is an outpatient, minimally invasive procedure; hemorrhoidectomy is an operative excisional treatment.	Hemorrhoidectomy provided better long-term clinical outcomes, higher quality-adjusted life-years, and lower recurrence, but with higher hospital and societal costs.	[70]
Rubber band ligation vs. sclerotherapy	Systematic review and meta-analysis	Symptomatic hemorrhoidal disease, mainly grade I–III	RBL induces ischemic necrosis and fixation; sclerotherapy induces chemical inflammation, fibrosis, and vascular obliteration.	RBL showed better control of prolapse and bleeding and higher patient satisfaction, but caused more postprocedural pain; recurrence was similar between procedures.	[71]
Rubber band ligation vs. sclerotherapy	Multicenter non-inferiority randomized trial protocol	Grade I–II hemorrhoids	RBL uses three rubber bands; sclerotherapy uses polidocanol injection.	The study is designed to compare symptom reduction, recurrence, complications, patient experience, number of treatments, and sick leave; results are not yet reported in this protocol.	[73]
Laser hemorrhoidoplasty vs. rubber band ligation	Randomized trial	Grade II hemorrhoids	LHP uses intralesional laser energy to shrink hemorrhoidal tissue; RBL applies elastic bands at the hemorrhoidal base.	LHP resulted in less postoperative pain, less bleeding, less anal distension, and faster return to normal activities; recurrence did not differ significantly at follow-up.	[74]

**Table 5:** Current guideline and guidance positions on radiofrequency-based techniques for hemorrhoidal disease.

Guideline/Organization	Year	RF Technique Addressed	Position on RF Techniques	Relevance for This Review
NICE interventional procedures guidance/HealthTech guidance	2017	Radiofrequency treatment for hemorrhoids, including intralesional probe-based treatment and surface ball-electrode treatment	The evidence on safety and efficacy is considered inadequate in quantity and quality, and the procedure should be used only with special arrangements for clinical governance, consent, audit or research.	RF treatment is recognized as a specific interventional procedure, but it should be presented cautiously and not as a fully established standard treatment.
SICCR consensus statement	2020	Radiofrequency hemorrhoidectomy	Radiofrequency hemorrhoidectomy is described as a sutureless technique using a modified electrosurgical unit for tissue and vessel sealing, with less blood loss, postoperative pain and complications; the assigned level of evidence is 1 and the grade of recommendation is B.	SICCR supports radiofrequency mainly in the context of radiofrequency hemorrhoidectomy, not specifically Rafaelo-type intralesional RFA.
ESCP guideline	2020	No specific radiofrequency or Rafaelo-type recommendation identified	The ESCP guideline provides a multidisciplinary international framework for hemorrhoidal disease management, but the terms “radiofrequency” and “Rafaelo” are not included in the guideline text.	Rafaelo-type RFA should be described as an emerging technique that remains outside the main ESCP treatment algorithm.
ASCRS clinical practice guideline	2024	No specific RFA or Rafaelo-type recommendation identified	ASCRS recommends dietary and behavioral modification as first-line therapy, office-based procedures for most grade I–II hemorrhoids and selected grade III hemorrhoids, and excisional hemorrhoidectomy for selected external or combined internal–external grade III–IV disease; “radiofrequency” is not included in the guideline text.	In relation to ASCRS guidance, RFA should be positioned as selectively used or investigational rather than guideline-established standard therapy.
AGA clinical practice update	2026	No specific RFA or Rafaelo-type recommendation identified	AGA best practice advice supports dietary and lifestyle treatment, anoscopy before treatment, rubber band ligation and infrared coagulation as office-based procedures, and surgical referral for grade III disease failing banding or grade IV disease.	Recent gastroenterology guidance supports conventional office-based escalation before surgery and does not define a standard role for RFA.
Japanese Practice Guidelines for Anal Disorders	2017	No specific RFA or Rafaelo-type recommendation identified	The Japanese guideline uses Goligher classification to guide treatment and discusses drug therapy, office-based treatment and surgery, but does not provide a specific recommendation for RFA.	RFA should be discussed separately from the established Japanese guideline-based treatment pathway.

## Data Availability

No new data were created or analyzed in this study.
